# Effect of tetramethylpyrazine on tibial dyschondroplasia incidence, tibial angiogenesis, performance and characteristics via HIF-1α/VEGF signaling pathway in chickens

**DOI:** 10.1038/s41598-018-20562-3

**Published:** 2018-02-06

**Authors:** Khalid Mehmood, Hui Zhang, Kun Li, Lei Wang, Mujeeb Ur Rehman, Fazul Nabi, Muhammad Kashif Iqbal, Houqiang Luo, Muhammad Shahzad, Jiakui Li

**Affiliations:** 1 College of Veterinary Medicine, Huazhong Agricultural University, Wuhan, 430070 P. R. China; 2 College of Animals Husbandry and Veterinary Medicine, Tibet Agricultural and Animal Husbandry University, Linzhi, Tibet 860000 P. R. China; 3 University College of Veterinary & Animal Sciences, The Islamia University of Bahawalpur, Bahawalpur, 63100 Pakistan

## Abstract

Tibial dyschodroplasia (TD) is a most common pathological condition in many avian species that is characterized by failure of growth plate (GP) modeling that leads to the persistence of avascular lesion in the GP. Tetramethylpyrazine (TMP) is widely used to treat neurovascular disorders and pulmonary hypertension, but no report is available about promoting effect of TMP against TD. Therefore, a total of 210 broiler chicks were equally divided into three groups; Control, TD and TMP. During the experiment mortality rate, chicken performance indicators (daily weight, average daily feed intake, average daily weight gain and feed conversion ratio), tibia bone indicators (weight, length, width of tibial and the size of GP) in addition to gene expression of HIF-1α and VEGF were examined. The results showed that TMP administration restore the GP width, increase growth performance, and mitigated the lameness in broiler chickens. The expression of HIF-1α and VEGF increased significantly in TD affected thiram induced chicks. Whereas, TMP treatment down-regulated HIF-1α and VEGF genes and proteins expressions. The present study demonstrates that the TMP plays an important role in angiogenesis during the impairment and recovery of GP in TD via regulation of the HIF-1α/VEGF signaling pathway in chickens.

## Introduction

Tibial dyschondroplasia (TD) is a tibiotarsal bone disorder in chickens which is characterized by avascular and non-mineralized growth plate (GP) and is recognized as lameness and abnormal differentiation of chondrocytes in avian species^1–3^. TD is widely occurred in fast growing birds, especially in chicken and turkey worldwide^1,4,5^. The lesion is characterized by endochondral ossification suffocate, tibial metaphyseal cartilage cell proliferation, and a mass of avascular cartilage in the metaphysis of the proximal ends of the tibiotarsus^6–11^. Clinically TD is manifested by depressing of spirit, decrease physical activity, less eating and drinking, standing difficulty, gait inflexible, movement disorders or wings support walking, skeletal malformations and growth performance degradation^2,3^.

A recent statistics showed that almost 30% of bone disease was TD in chickens and turkeys, while it caused more than 10% morbidity in China that placed serious economic losses to the poultry industry^12–14^. Normally, the clinical and subclinical rate of TD in chickens result in decrease in disease resistance and production performance, induce breast cyst and osteomyelitis, which leads to cruelty and animal welfare issues and reduces the carcass quality in chickens^7,15,16^. TD is a metabolic diseases and nutritional disorder that affect broiler production^17^. Although these problems are of great concern for researchers worldwide, but the underlying etiology of TD remains unknown^8,18,19^. As TD cause massive loss to broiler production worldwide, therefore, close attentions is required to identify its etiology and occurrence.

The capillary invasion mediated by vascular endothelial growth factor (VEGF) is a key mechanism for the precise coupling of chondssrogenesis and osteogenesis that determines the rate of bone growth and is a prerequisite for bone formation^8,9,20–23^. Any changes in this balance might induce pathological conditions, as exemplified by the large numbers of human chondrodysplasias and animal transgenic models^24,25^. Rath *et al*.^8^ studied that the occurrence of TD is due to the disruption of genes encoding VEGF receptors followed by subsequent endothelial cell death, which compromises vascularization and the removal of dead chondrocytes and leads to TD lesions. Recent studies show that production of VEGF by the hypertrophic chondrocytes is regulated by hypoxia and by hypoxia-inducible factor-1 (HIF-1α) that plays major roles in the prevention of TD^9,26–30^.

Tetramethylpyrazine (TMP) is an important active ingredient of traditional Chinese medicines, and it is identified with several molecular targeting properties^31^. In China it is used to treat ischemic stroke, neurovascular disorders, pulmonary hypertension and obstructive pulmonary diseases due to its effectiveness and low toxicity^31–33^. It has also been found that TMP has a protective effect in vascular growth via VEGF and its upstream factor HIF-1α^34–36^. Considering the characteristics of TMP, we hypothesized that it can prevent TD by tibial angiogenesis. Therefore, this study was conducted to evaluate the effects of TMP on tibial dyschondroplasia incidence, tibial angiogenesis, performance, and characteristics via regulation of the HIF-1α/VEGF signaling pathway in broiler chickens.

## Results

### TMP prevented the mortality in chickens

**Figure 1:**
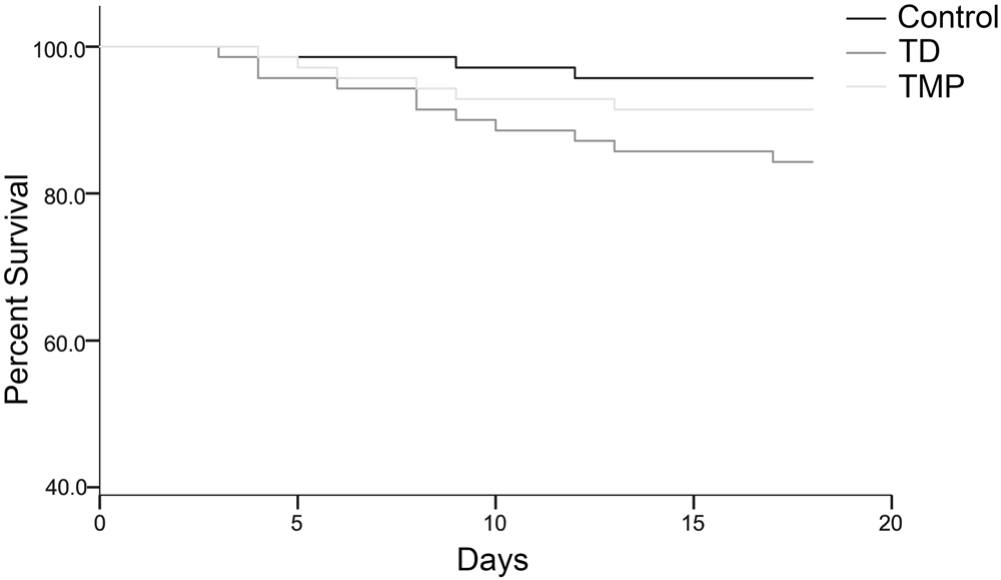
The percentage of survival among control, TD and TMP groups of chickens during the experiment period. The curve was generated by using SPSS22.0.

The mortality was evaluated from day 1 to day 18 in each group. The results showed that mortality rate on day 7 and 10 were much increased in TD group as compared with control and TMP groups. Although, the TD was reduced after removing the thiram from day 14 to 18, but mortality was higher in TD group. However, after the administration of TMP on day 8, the mortality was decreased in TMP group compared with TD group but difference was not significant as shown in Table 1. The survival percentage of chickens among TD, TMP and control groups was shown in Fig. 1.

**Table 1:** The mortality rate among control, TD and TMP groups from day 1 to day 18.

Days	Control group (n = 70)	TD group (n = 70)	TMP group (n = 70)
^*^1–7	1	4	3
^#^8–10	1	4	2
11–14	1	2	1
15–18	0	1	0
**Total**	**3**	**11**	**6**

Chi-square analysis for total number dead and alive chickens (χ^2^ = 5.416, df = 2, p = 0.066). ^*^Thiram was given to TD and TMP groups from day 4 to day 7. ^#^TMP was administered to TMP group from day 8 to day 18.

### Performance parameters analysis of chickens

**Figure 2:**
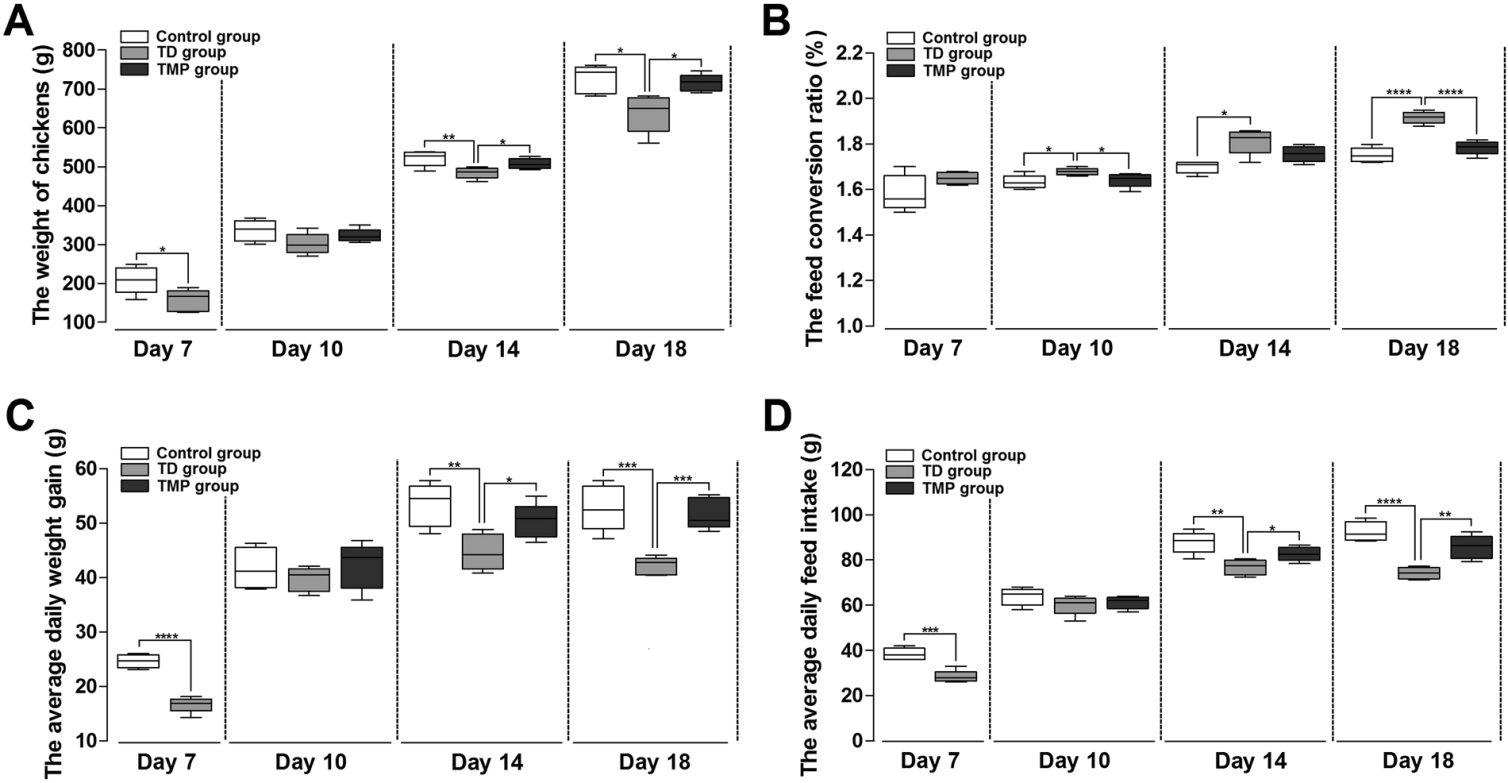
Overall performance parameters analysis of chickens among control, TD and TMP groups on various days 7, 10, 14 and 18. (**A**–**D**) correlation analysis among weight of chickens, average daily feed intake, average daily weight gain and feed conversion ratio were recorded using SPSS version 19.0. The data are expressed as the mean ± SD. *P < 0.05, **P < 0.01, ***P < 0.001, ****P < 0.0001.

The results indicated that thiram induced TD group show significantly decrease in production parameters of chickens as compared to control group. The daily weight, average daily feed intake and average daily weight gain was significantly reduced in TD group than control group after the treatment of thiram group on 7 day. On 10 day, there was no significant difference in control, TD and TMP groups. However, administration of TMP in TD-affected chickens significantly increased the daily weight, average daily feed intake and average daily weight gain of chickens especially on day 14 and 18 as compared with TD afflicted group. Meanwhile, feed conversion ratio (FCR) of chickens was poor on day 10, 14 and 18 in TD group as compared with control and TMP groups (Fig. 2).

### Effect of TMP on TD incidence

**Figure 3:**
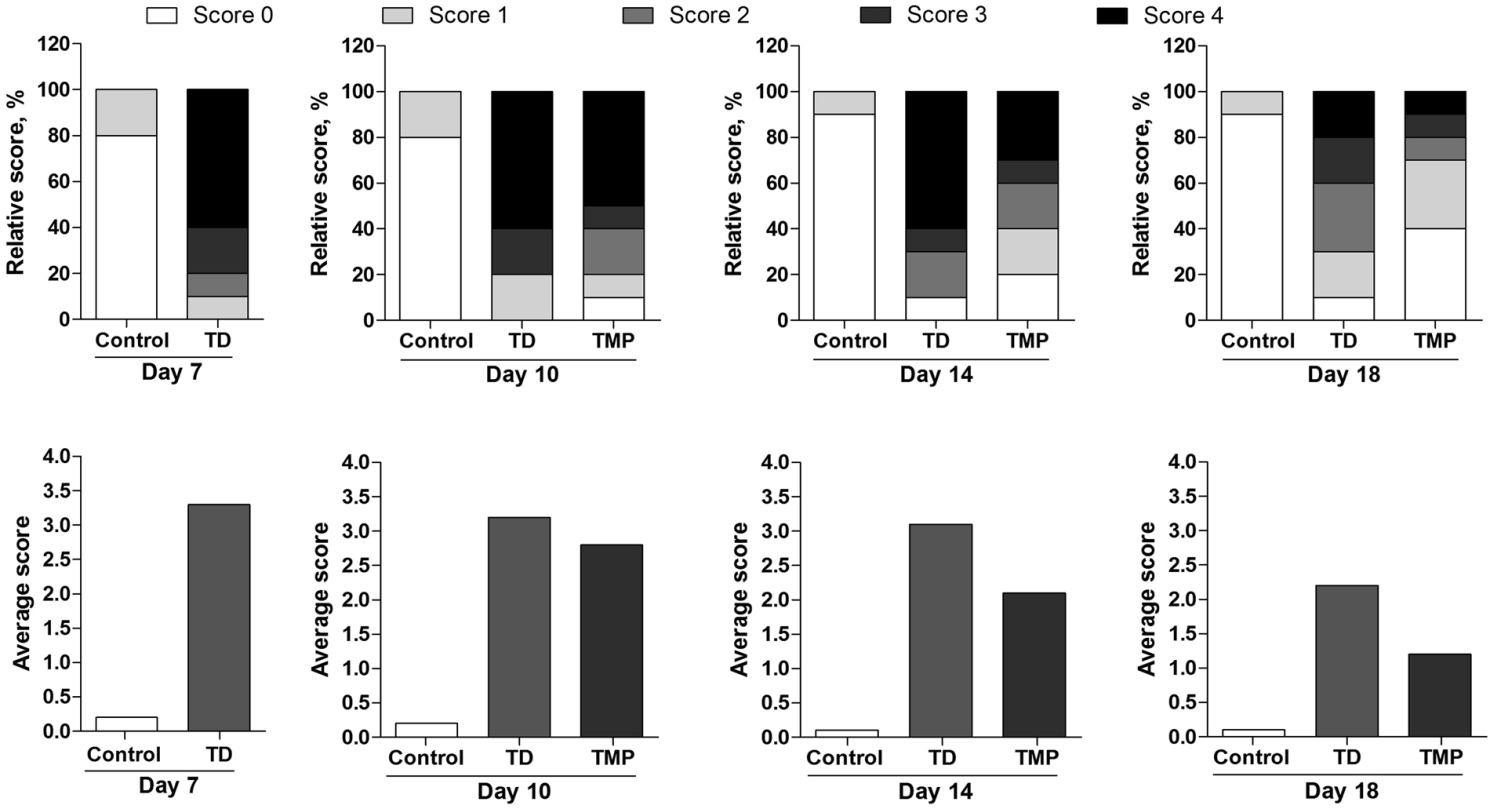
Effect of thiram on tibial dyschondroplasia (TD) incidence and effect of TMP on TD score in chickens. All tibial growth plates were dissected and scored for TD severity on day 7, 10, 14 and 18 for average and relative TD score. TMP gave significant effect especially on day 14 and 18.

The results showed, thiram can induce TD easily in TD group, and more than 90% chickens were found lame after 4 day of thiram administration. The TD score was higher in TD group as compared than control and TMP groups. In control group, tibia had almost normal phenotype and relative TD score was less. However, TMP group showed that chicken restored the TD pathogenesis after the administration of TMP and almost 80% chickens were found healthy because their relative TD score was less than 2. The average TD score was more than 3 in TD group on days 7, 10 and 14, while it was more than 2 at the end of experiment on day 18. However after the treatment of TMP to chickens, the average TD score was significantly decreased in TMP group on day 10, 14 and 18 (Fig. 3).

### The overall tibial parameters analysis

**Figure 4:**
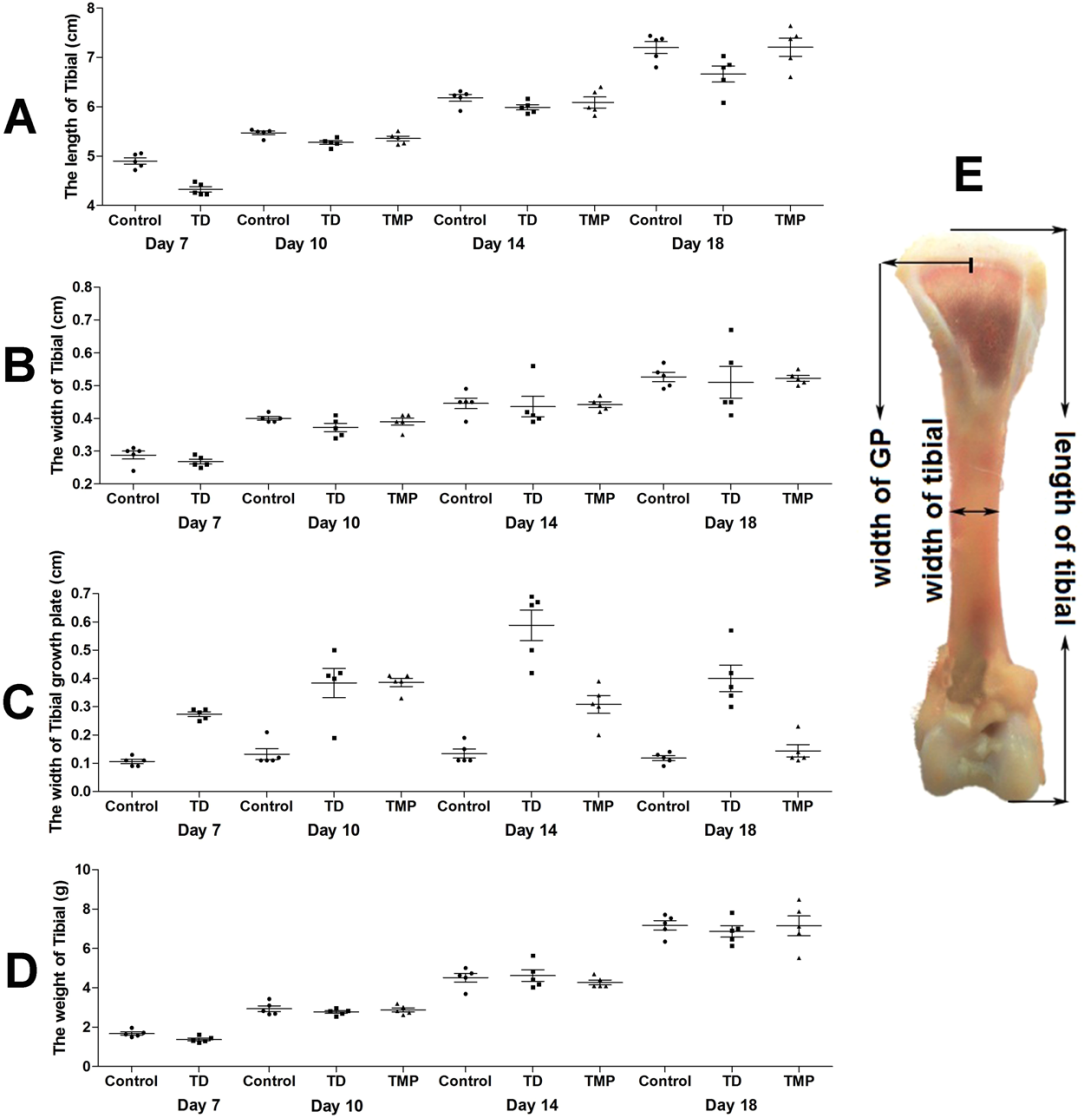
The overall tibial parameters analysis among control, TD and TMP groups on various days 7, 10, 14 and 18. (**A**–**D**) Correlation analysis among length of tibia, width of tibia, weight of tibia and width of tibial growth plate were recorded with Pearson test. (**F**) Measurement of length of tibia, width of tibia and width of tibial growth plate. The difference was not significant among all the parameters except width of tibial growth plate (P < 0.05).

The changes of tibia parameters (length, width, weight and width of GP) in chickens were recorded among all the groups on days 7, 10, 14 and 18. Overall, length, width and weight of tibia bone were more in control and TMP group chickens than TD group on days 10, 14 and 18, but the difference was non-significant among these three groups. However, the width of proximal GP of thiram-induced TD chickens were markedly enlarged (P < 0.05) from day 7 to day 14 in TD group as compared to control group chickens. Whereas, administration of TMP significantly decreased the GP width in TMP group on days 10, 14 and 18. The chickens retain the ability to stand and walk properly in TMP group. In contrast, thiram-induced TD had a more apparent impact on the growth-plate width as compared with control group and TMP group. In addition, the chicken has self-healing ability in TD group especially after day 14. However, the TD restored slowly as compared with the TMP group (Fig. 4).

### Clinical observation of thiram-induced TD and TMP chickens

**Figure 5:**
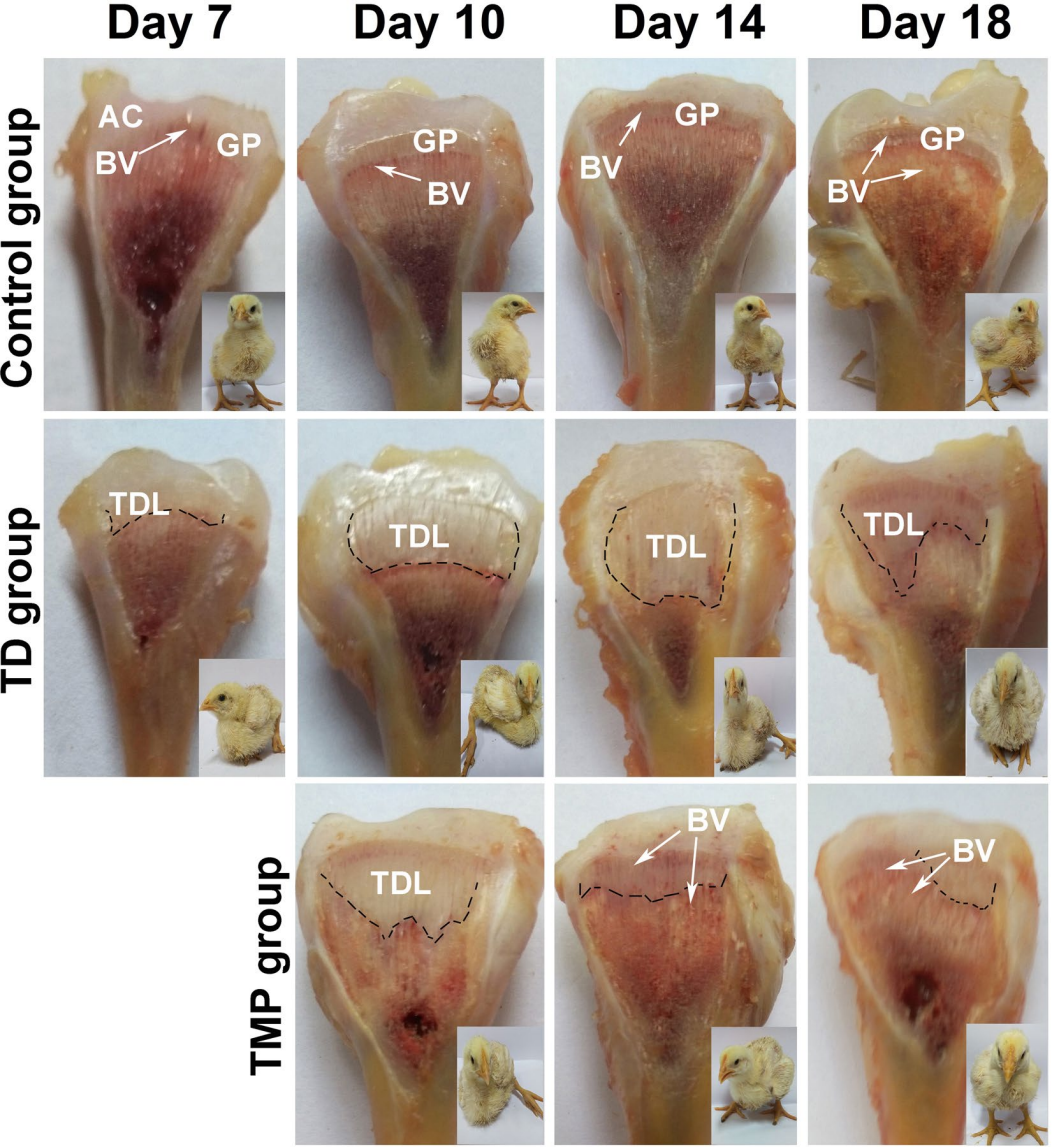
The morphological analysis of growth plates of control, thiram, and TMP treated chickens on day 7, 10, 14 and 18. Extended GP was compared in TD group as compared with control and TMP group. AC = articular cartilage, BV = blood vessel, GP = growth plate, TDL = tibial dyscondroplasia lesion.

The chickens started showing signs of disease on day 4 post hatch. Almost >90% of chicks were showing lameness on day 7 post hatch in TD group. The visual examination revealed the depression, poor body condition of chickens in thiram-induced TD with weakness, lameness and feeding difficulties. However, in TMP treatment group the lameness was reduced, which continued to subside until day 18 with only 2 percent birds showing minor signs of disease as compared to the TD groups. The TMP treatment chickens regained their ability to stand and walk properly (Fig. 5). TD afflicted GP revealed an opaque, avascular cartilaginous mass and GP enlargement on days 7, 10, 14 and 18 as compared to control group. The TMP treated group revealed a prominent difference in the size and shape of GP, it was markedly reduced and reverted to the control levels and the incidence of lameness diminished significantly as compared to TD affected birds.

### Histological examination of the tibial growth plates

**Figure 6:**
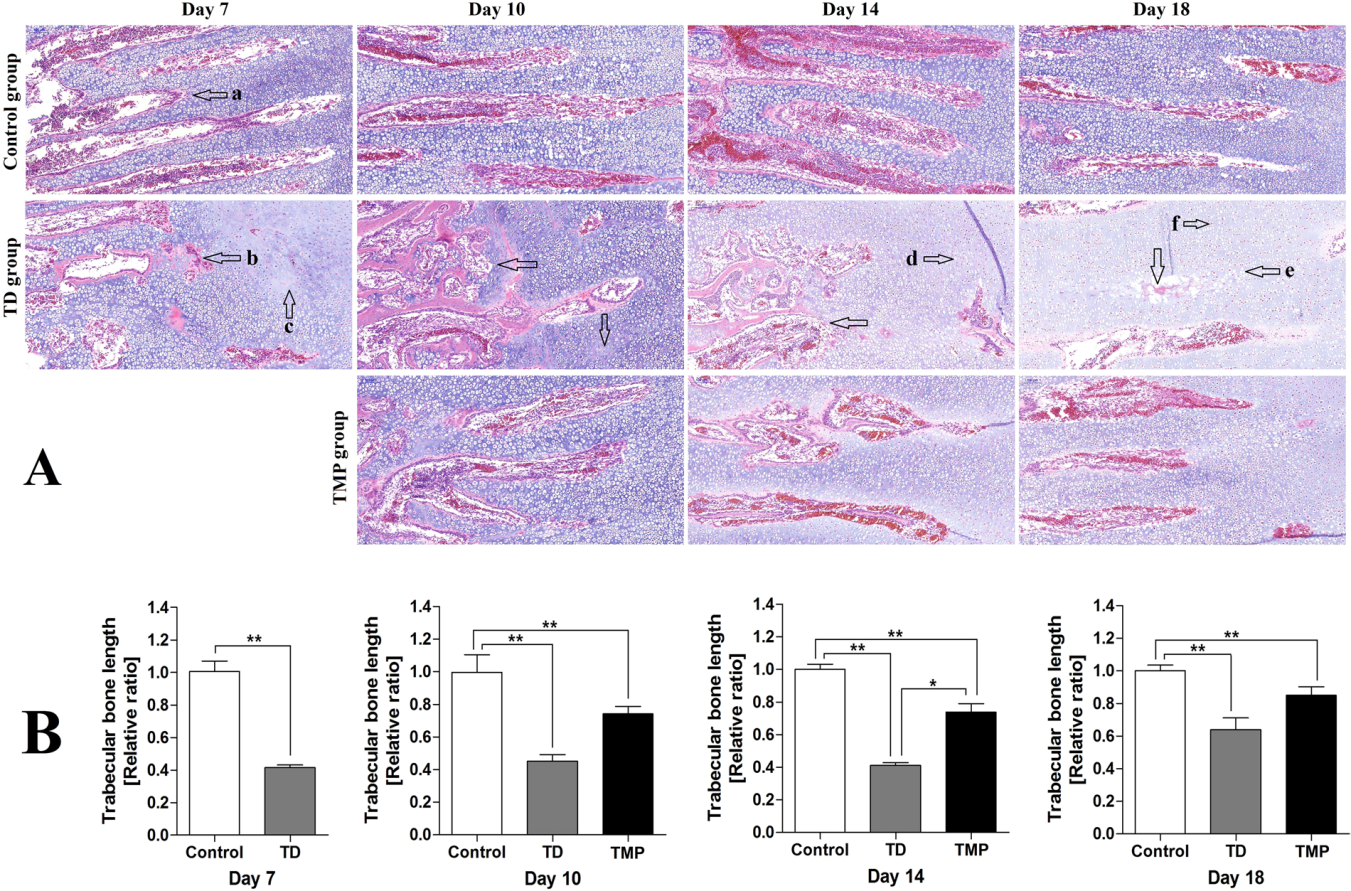
H&E stained histopathology micrograph depicts of blood version in tibial bone. (**A**) Representative images stained with H&E in tibias. (a) Normal trabecular bone; (b) broken trabecular bone; (c, d) the hypertrophy zone cytolysis; (e, f) the nucleus dissolved and migrated. (**B**) The comparison of trabecular bone length among different groups at various days. The data are expressed as the mean ± SD. *P < 0.05, **P < 0.01.

Histology of tibial GP showed well-conserved columns and surrounded with large number of blood vessels in control group. However, in TD group a large number of cell death, empty cartilages, disordered arrangements of chondrocytes, degradation was found in growth plate. After TMP treatment, angiogenesis was observed and it restored regular columns of cells surrounded by blood vessels in tibia than TD group (Fig. 6A). The trabecular bone length was less in TD group chicken than control group on 7 day. Whereas, after TMP administration there was prominent enlarge in the length of trabecular bone on day 10, 14 and 18 compared with TD group chickens (Fig. 6B).

### Immunohistochemistry of Tibial growth plates

**Figure 7:**
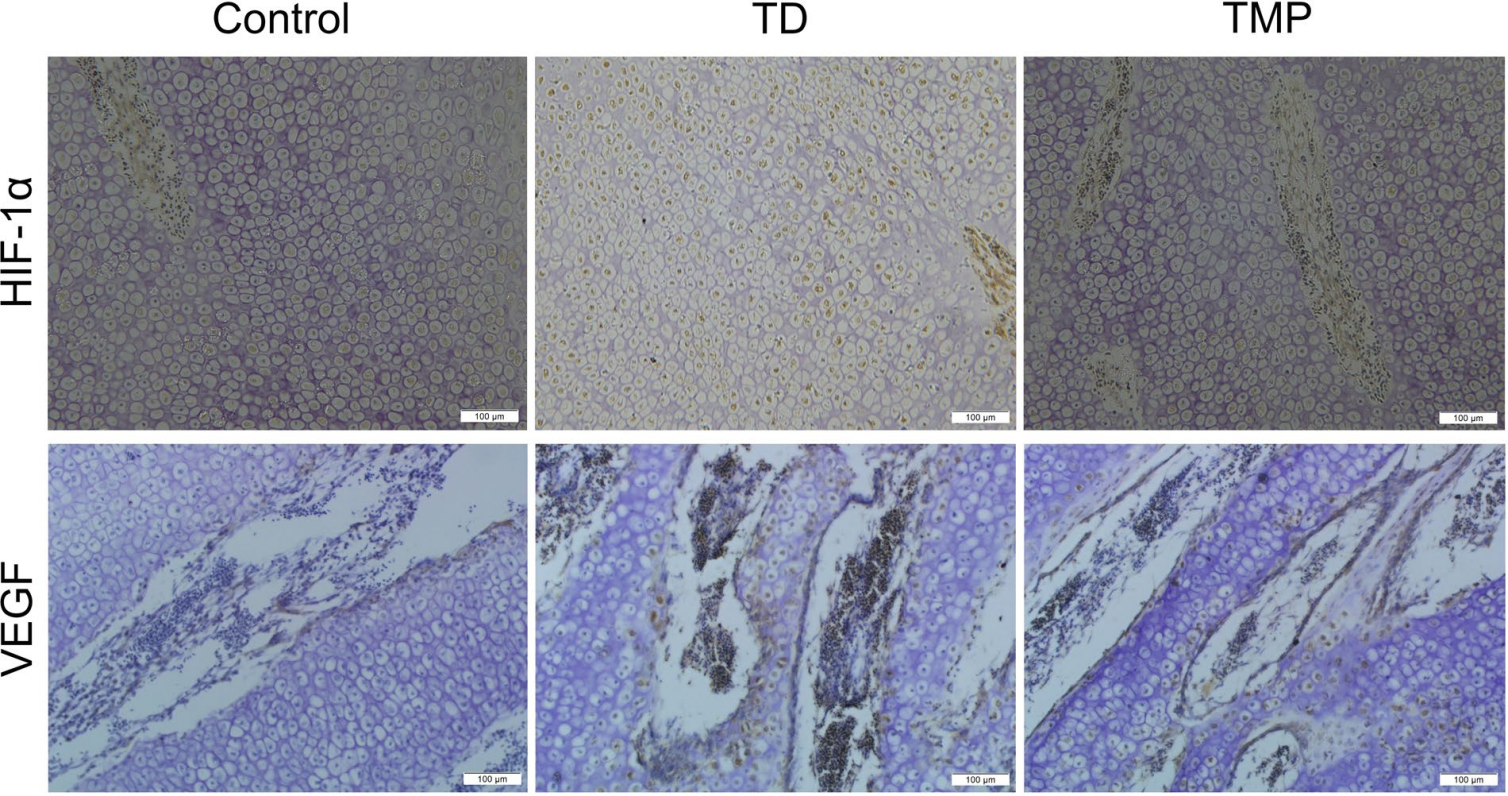
Immunohistochemical localization of HIF-1α and VEGF in control, thiram and TMP groups. TD group has more localization of both HIF-1α and VEGF than control and TMP groups.

The expression of HIF-1α and VEGF antibody in tibial growth plate was checked by immunohistochemistry. The HIF-1α expression appeared in more number of cells in thiram group compared to control. Whereas, the expression of HIF-1α was decreased in TMP group compared to TD group. The VEGF localization was more in thiram group than control group, as more cells were positively stained with VEGF antibody in thiram fed chickens. The TMP administration showed less localization of VEGF compared to TD group (Fig. 7).

### The expression and protein level of HIF-1α/VEGF genes in growth plate

**Figure 8:**
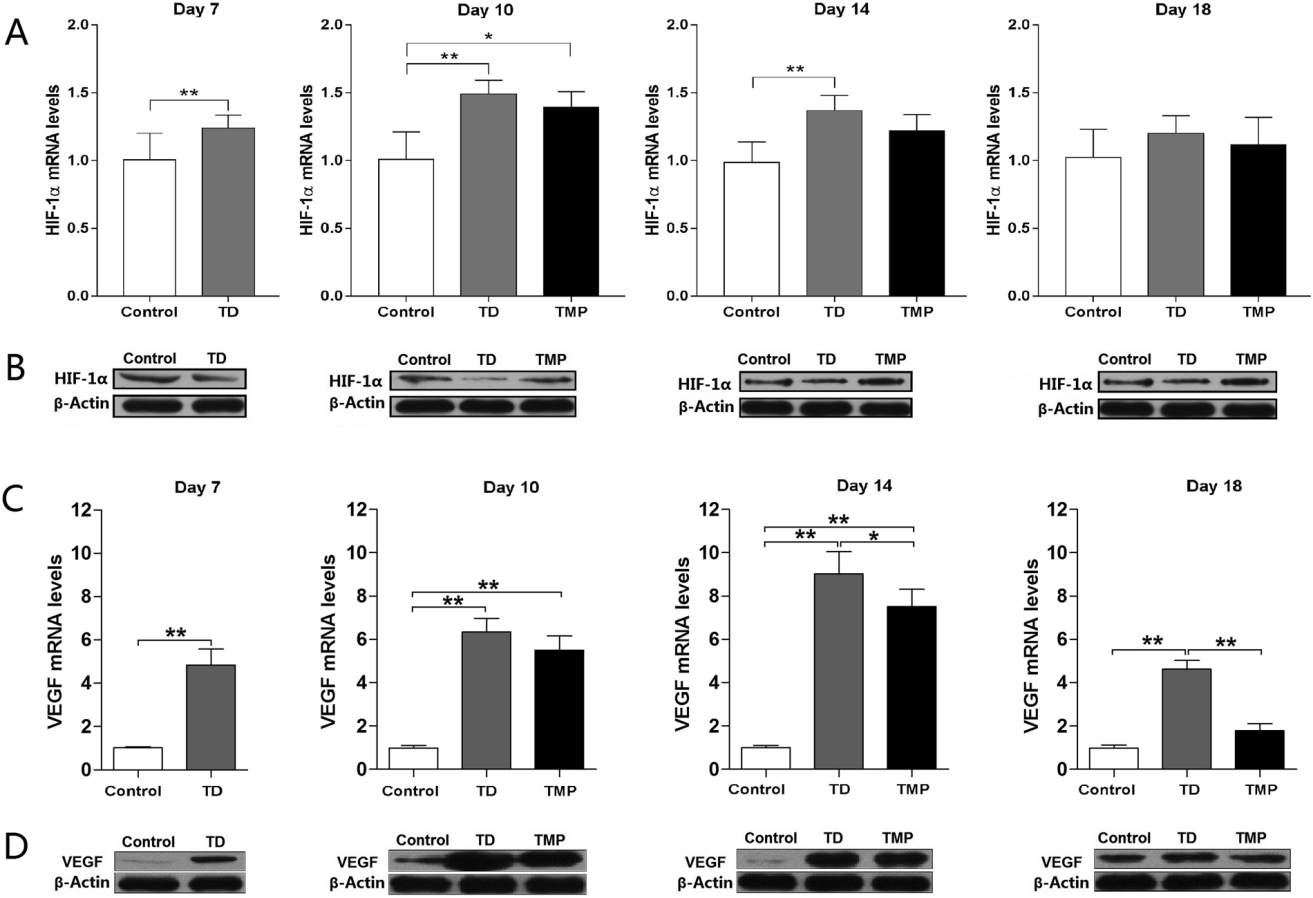
Real-time quantitative PCR and Western blotting analysis of HIF-1α and VEGF in tibial growth plate on various days 7, 10, 14 and 18. (**A**) The mRNA levels of HIF-1α were detected by RT-qPCR on various days in control, TD and TMP groups. (**B**) Western blotting was performed to detect the protein levels of HIF-1α on various days in control, TD and TMP groups. (**C**) The VEGF mRNA levels were detected by RT-qPCR in control, TD and TMP groups. (**D**) Western blotting was performed to detect the protein levels of VEGF on various days in control, TD and TMP groups. Correlation coefficient = target gene (average optical density)/reference gene (average optical density). The results are expressed in arbitrary units as the means ± SEM. The mRNA levels from the control group were normalized to a value of 1. GAPDH and β-actin were used as the loading control in RT-qPCR and western blotting respectively. *P < 0.05, **P < 0.01.

The mRNA expression and protein profile of genes involved in the GP of chickens was examined on day 7, 10, 14 and 18. The HIF-1α and VEGF genes and protein expression level were explored to find the relationship between gene expressions in TD and normal GP. The results revealed that HIF-1α expression was significantly up regulated from day 7 to 14 in TD afflicted group as compared to control group. Compared with TD group, TMP treatment significantly decreased the HIF-1α expressions levels in growth plate cartilage. The mRNA expression of VEGF was up regulated in TD group during the study period. However, the TMP administration reduced the VEGF expression from day 14 and to 18. Furthermore, western blotting analysis results were also parallel to gene expression results (Fig. 8).

## Discussion

Tibial Dyschondroplasia (TD) is an important cartilage metabolic disease with leg problem in fast growing birds that disturbs the proximal tibial growth plate^1,4^. This dull white avascular and non-calcified bone leads to locomotion problems along with 30% economical loss to the broiler flocks^12–14^. Since the discovery of TD, many factors have been studied to rule out the exact cause of this economically important disease. Although, various protocols have been adopted to minimize the incidence of TD, but the mechanism(s) and underlying its pathogenesis is still unknown. TD is characterized by non-vascularized cartilage, a distended GP, tibial bone deformation and lameness^2–4^. Tetramethylpyrazine is an important active traditional Chinese medicine, it can change the protein and mRNA expression levels of VEGF and HIF-1α^34–36^. Experimentally, it has also been reported to inhibit hypoxia-induced pulmonary endothelial leakage in rat pulmonary microvascular endothelial cells monolayers. It provided a novel evidence for its protective effect that is associated with the moderation of the hypoxia-induced HIF-1α factor and VEGF expression^34,35^.

Recent studies reported that TD can induce decrease in physical activity level, no-eating and drinking, standing difficult, gait inflexible, movement disorders, which will reduce body weight gain, average daily feed intake, average daily weight gain and increase FCR^2–5^. In our findings daily weight, average daily weight gain and average daily feed intake was increased compared to TD group with the use of TMP, especially on day 14 and 18. The FCR was also good as compared with TD group. TD chicks faced obstacles in walking, uncoordinated body movement, unable to stand, reduced feed intake, poor weight gain, and lower FCR, which seriously affect the production performance and growth indicators in broilers. Our results indicated that TMP was effective to the recovery TD rate and degree of growth performance found improved after TMP treatment. Genin *et al*.^37^ showed that the GP width was not changed in normal chickens, while in TD-afflicted chickens the enlarged GP, TD lesions and lameness happened. Current study showed that the mortality rate were significantly higher in TD group as compared to control group. However, the morality decreased with the use of TMP, which indicated that the TMP was effective to treat TD.

In TD, proliferative zone or hypertrophy zone of growth plate had a large number of cell death, appeared empty, mixed together or disordered arrangement, degradation and irregular hypertrophy zone was found due to miscellaneous arrangement of cell in proliferative and hypertrophic zone^4,15^. Shirked calcified area, vascular bending, hypertrophy of cartilage cell irregularly, nuclear pyknosis which leave the empty pit of cartilage, interstitial cells increased, bone resorption was slow and narrow trabecular bone^8,10^. In our study, we found growth plate cartilage cell proliferation and hypertrophy zone cell differentiation make the tibia avascular, less extracellular matrix and non-calcified, which lead to the failure of angiogenesis in the tibial growth plate and thereby resulting in the transportation hurdles of nutrition to chondrocyte^4^. Ultimately, cartilage in the zone of mineralization and calcium deposition fails to complete the calcification^4,38^.

Vascular endothelial growth factor is an antiparallel dimmer of glycoprotein with multiple biological functions^39^. Previous reports showed that VEGF is not only a fundamental mediator of physiologic and pathophysiologic angiogenesis but also plays a critical role in regulation of vascular invasion, matrix degradation and remodeling, differentiation, maturation, survival and functional activity of chondrocyte, osteoblast, osteoclast by autocrine/paxacrine way during endochondral and membranous ossification^40,41^. Our study found an increase in mRNA and protein expression level of VEGF in TD chickens. Thiram appeared has pro-angiogenic acivity^8^ and it can up regulate VEGF^4,16^. Previous studies have shown VEGF is produced by hypertrophic chondrocytes^35^, which is regulated by HIF-1α^36^. The capillary invasion mediated VEGF have important role during chondrogenesis and osteogenesis^4,8^, and any change in this balance and mechanism may induce pathological conditions in broiler chicken. Our findings of up regulation of VEGF expression are concomitant with previous reports^4,16,42,43^. Dan *et al*.^14^ and Genin *et al*.^37^ stated that TD endorsed the change in VEGF signaling pathways and abnormal chondrocyte differentiation. The angiogenic activity of TMP was substantiated by assessing VEGF gene and protein expression. TMP was effective to reduce the localization of VEGF, and HIF-1α in immunohistochemistry of lungs^35^. Previous studies also reported similar results that TMP decreased the protein and mRNA expression levels of VEGF and HIF-1α^34–36,44^.

In conclusion, TMP have effective performance on tibial angiogenesis and prevention of TD incidence in broiler chickens. The present study demonstrated that ameliorative effect of TMP in broiler chickens via regulations of HIF-1α/VEGF signaling pathway. This is the first study that explains the effect of TMP in thiram-induced TD. Therefore, HIF-1α/VEGF signaling pathway and targeted therapy are recommended here for the control and treatment of tibial dyschondroplasia.

## Materials and Methods

### Animal ethics and welfare

All the experiments were approved and reviewed by Institutional Animal Welfare and Research, Ethics Committee guidelines of Huazhong Agricultural University, Wuhan, China (Approval number: 31273519). All animal experiments and procedures were conducted under the relevant procedures of Proclamation of the Standing Committee of Hubei People’s Congress (PSCH No.5), China.

### Chicken management and experimental design

Two hundred and ten 1-day-old Arbor Acres (AA) broiler chickens (weighing 48 ± 6 g) were grown under recommended standard temperature. The chickens were given standard diet ad libitum for 3 days as suggested by the National Research Council^45^. On day 4, the chickens were randomly distributed into three groups; control group (n = 70) administered standard diet. Whereas, thiram group (n = 70) and TMP group (n = 70) received the same diet with the addition of tetramethylthiuram disulphide (thiram) @ 50 mg/kg of feed from day 4-post hatch to day 7 to induce TD. On the day 8, TD group was given standard diet without thiram just like control group, but TMP group was administered TMP @ 30 mg/kg/day till the end of the experiment^46–48^.

### Morphological, production parameters analysis and sample collection

All groups were raised for 18 days and the number of morbidity, mortality, lame birds, daily weight gain and feed intake in each group was recorded on daily basis. During the experiment period, 15 chickens were randomly selected to sacrifice by cervical dislocation on days 7, 10, 14 and 18 from each group. After the euthanizing, the tibial bones were measured for morphological examination including weight, length, and width of tibial bone and size of tibial GP by an electronic balance and Digital Calipers (SATA91511, TATA Company, Shanghai, China), respectively. The TD score was calculated in all the groups as described by Pines *et al*.^10^. Then some of the tibial bones (n = 10) were fixed in 4% paraformaldehyde (hematoxylin & eosin staining and immunohistochemistry) and other tibial bones (n = 20) were immediately frozen in liquid nitrogen and then stored at −70 °C for further analysis (Western blotting and RT-qPCR analysis).

### Hematoxylin & Eosin (H&E) staining and Immunohistochemistry

The tibiotarsal bone (n = 10) samples were stained with H&E staining according to Mehmood *et al*.^49^.

For immunohistochemical, the slides were washed in PBS and peroxidase blocking solution (Boster, Wuhan), and then incubated with primary antibody for VEGF (1:1000), and HIF-1α (1:500) overnight at 4 °C. After washing with PBS, it was incubated with a secondary antibody (1:200) in the dark for 2 h at 25 °C. The Image-Pro® Plus 6.0 was used to analyze the blood vessel area.

### Quantitative-real time PCR (RT-qPCR)

Total RNA was obtained from primary tibial GP (n = 10), while RNA was extracted using Trizol reagent (Invitrogen, Carlsbad, California, USA), and converted into cDNA by using first-strand reverse transcription cDNA kit (Tian Gen, China) according to manufacturer’s instructions. Based on the published HIF-1α and VEGF gene sequences in the GenBank database, the primers were designed with the help of Primer Premier Software (version 5.0) and synthesized by Wuhan Qingke biotechnology Co., Ltd (Wuhan, China) as showed in Table 2. The RT-qPCR was performed in quadruplex with Step One-Plus ^TM^ Real-Time PCR System (Applied Biosystems). The RT-qPCR reaction system was performed according to our previous study^50,51^. All the reaction mixture was normalized against the reference gene GAPDH.

**Table 2:** Primers used for the Quantitative Polymerase Chain Reaction (qPCR).

Genes	Accession number	Primer sequence (5′-3′)	Product size (bp)	Tm (°C)
*VEGF*	XM_019612783	F: AAAGCGAGGAAAGGGGAAGG	94	55
		R: TCTCCTCTCTGAGCAAGGCT		
*HIF-1a*	Q16665	F: TGAGAGAAATGCTTACACACAG	263	56
		R: TGATGGGTGAGGAATTGGTTCAC		
*GAPDH*	XM_019960295	F: CCTTCATTGACCTTCACTACATGGTCTA	127	58
		R: TGGAAGATGGTGATGGCCTTTCCATTG		

### Western Blot analysis

Growth plates (n = 10) of individual tissues were homogenized in PBS and then put at 4 °C for 2 h and after that it was centrifuged at 14,000 rpm for 10 min. The equal amounts of protein were separated by SDS-PAGE on 10% gel of polyacrylamide. The membrane was incubated in 5% skim milk for 1.5 h and then treated with primary antibody (rabbit monoclonal anti-HIF-1α or mouse monoclonal anti-VEGF antibody, Abcam, 1:1000 dilutions) at 4 °C overnight. The membranes were washed 3 times with tris-buffered saline (containing 0.1% Tween 2.0) for 5 min each, and then incubated with goat anti-mouse secondary antibody (Abcam, 1:3000 dilution) for 30 min at room temperature. Then membranes were washed with tris-buffered saline and image was captured with an imaging system (UVP, Upland, CA, USA).

### Statistical Analysis

All analyses were performed to evaluate comparison among three groups using SPSS Statistics software (SPSS Version 19.0) by one way ANOVA and student t-test and presented as means ± standard error of means (SEM). *P* < 0.05 was considered significant.
